# Total Barley Maiya Alkaloids Prevent Increased Prolactin Levels Caused by Antipsychotic Drugs and Reduce Dopamine Receptor D2 *via* Epigenetic Mechanisms

**DOI:** 10.3389/fphar.2022.888522

**Published:** 2022-07-05

**Authors:** Yu-Ling Cao, Li -Zhu, Hong Zhang, Jun-Hua Meng, Hua-Jun Wu, Xiong Wang, Jin-Hu Wu, Ji-Li Zou, Mao-Sheng Fang, Jing An, Yong-Gang Chen

**Affiliations:** ^1^ Pharmacy Department of Wuhan University Tongren Hospital (The Third Hospital of Wuhan), Wuhan, China; ^2^ Medical College of Wuhan University of Science and Technology, Wuhan, China; ^3^ Department of Psychiatry, Wuhan Mental Health Center, Wuhan, China

**Keywords:** antipsychotic drugs, total barley maiya alkaloids, dopamine D2 receptor, DNA methylation, prolactin

## Abstract

**Background:** The dopamine D2 receptor (DRD2) plays an important role in the increased prolactin (PRL) levels associated with the pathogenesis of antipsychotic drugs (ADs). Elevated prolactin levels can affect people’s quality of life. Maiya alkaloids has been used to treat diseases associated with high PRL levels. Maiya, is a processed product of the mature fruits of Hordeum vulgare L. (a gramineous plant) after sprouting and drying and also a common Chinese herbal drug used in the clinic, is traditionally used to treat abnormal lactation, and is currently used clinically for the treatment of abnormal PRL levels.

**Aims:** Epigenetic mechanisms can be related to DRD2 expression. We investigated the role of DRD2 methylation in the induction of PRL expression by ADs and the mechanism underlying the effects of total barley maiya alkaloids (TBMA) on this induction.

**Methods:** The methylation rate of DRD2 in 46 people with schizophrenia who took risperidone was detected by MassARRAY sequencing. Humans were long term users of Ris. Seventy Sprague Dawley female rats were divided into seven groups. A rat model of risperidone-induced PRL was established, and the potential protective effects of TBMA and its components [e.g., hordenine (Hor)] on these increased PRL levels were investigated. The PRL concentration was detected by Enzyme-linked immunosorbent assay. PRL, DRD2, and DNA methyltransferase (DNMT1, DNMT3α, and DNMT3β) protein and mRNA expression were detected by western blotting and real-time polymerase chain reaction (RT-PCR), respectively. The positive rate of methylation in the DRD2 promoter region of rats was detected by MassARRAY sequencing.

**Results:** Clinical studies showed that the positive rate of DRD2 methylation associated with increased PRL levels induced by ADs was significantly higher than in the normal prolactinemia (NPRL) group. *In vivo* and vitro, TBMA and Hor inhibited this induction of PRL expression and increased DRD2 expression by inhibiting the expression of the DNMTs.

**Conclusions:** TBMA and hordenine increased DRD2 expression by inhibiting DNMT-dependent DRD2 methylation.

## Introduction

Schizophrenia is among the global most disabling health conditions (Lee et al., 2016) and affects about 21 million people worldwide (Vos et al., 2016; Li et al., 2018; Jung et al., 2021). People with schizophrenia have a life expectancy 15 years shorter than the general population (Pillinger et al., 2019; Lobo et al., 2021). Antipsychotic drugs (ADs) and psychological consultation are commonly used to treat schizophrenia. However, ADs can cause many side effects, particularly causing an abnormal increase in prolactin (PRL), with an incidence rate as high as 70% (Bostwick et al., 2009; Alosaimi et al., 2018). This increase is often accompanied by amenorrhea, galactorrhea, and infertility (Bostwick et al., 2009), seriously affecting the quality of life.

Previous studies focused on the pathogenesis of PRL increases induced by ADs (Huang et al., 2020). However, given the exacerbated severity of PRL increase caused by ADs, the epigenetic mechanisms involved in this pathogenesis have become an area of interest. DNA methylation is catalyzed by DNA methyltransferases (DNMTs) (Huang et al., 2020) and regulates a variety of biological and pathological processes (Stresemann et al., 2006; Hamidi et al., 2015). DNA methylation is mainly observed in CpG islands and strongly correlates with transcriptional suppression (Antequera and Bird, 1993; Horii and Hatada, 2016). During DNA methylation, methyl groups are transferred by DNA methyltransferases, including DNMT1, DNMT2, DNMT3α, DNMT3β, DNMT3C, and DNMT3L (Robertson, 2001; Barau et al., 2016). DNMT1, DNMT3α, and DNMT3β are the major DNA methyltransferases in animals, maintaining methylation on hemimethylated CpG sites (Borowczyk et al., 2009). DRD2, a regulator of transcriptional responses, modulates PRL expression in the pituitary gland. Therefore, DRD2 inactivation or mutations decreasing DRD2 mRNA or protein expression can result in PRL accumulation, activating PRL transcriptional programs (Céspedes, 2017; Gerra et al., 2021). The noncoding region of DRD2 contains CGG repeat sequences that provide a foundation for methylation. Previous studies revealed that CpG hypermethylation of DRD2 leads to gene inactivation and loss of function in alcohol-exposed rats (Gangisetty et al., 2015). Emerging evidence has demonstrated that DRD2 expression is tightly regulated by DNA methylation. Several studies have reported that the DRD2 methylation rate increases with decreasing DRD2 expression (Tan et al., 2021).

Risperidone (Ris) is a common antipsychotic drug used for treating schizophrenia; however, increases in PRL levels are common after long-term administration, leading to serious adverse reactions, such as amenorrhea, galactorrhea, anovulation and infertility (Zhu et al., 2021). In addition, opioids (Demarest et al., 2015), estrogen (Ni et al., 2021), and contraceptives (Taşkömür and Erten 2021) can increase PRL levels. We hypothesized that the increased PRL levels associated with these drugs and hormones might be related to DRD2 methylation because clinical data showed that AD-induced increases in PRL levels are associated with DRD2 methylation. In China, bromocriptine (Bro), a DRD2 agonist, is the first choice for the clinical treatment of the elevated PRL levels caused by ADs; however, this treatment is accompanied by hallucinations, conscious insanity, digestive system disorders, and other adverse reactions (Chen et al., 2017). Therefore, more suitable therapeutic drugs are needed.

Multiple studies have shown that maiya is an edible traditional Chinese medicine for treating digestive disorders and abnormal PRL levels with fewer side effects than bromocriptine. In a preliminary study, we extracted total barley maiya alkaloids (TBMA) from maiya and found that hordenine (Hor) accounted for 8.58% of the active components in the TBMA (Tao et al., 2021). Previous studies confirmed that TBMA and hordenine were the active ingredients reducing PRL and regulating lactation. In addition, we have shown that TBMA and hordenine can inhibit abnormal PRL secretion by upregulating DRD2 expression (Gong et al., 2021). However, the epigenetic mechanism by which TBMA inhibits PRL secretion and the increased PRL levels associated with ADs has not been clarified. The current study investigated the epigenetic mechanism of PRL inhibition by evaluating the active components in maiya (i.e., TBMA and Hor). To this end, we used a Ris-induced increased PRL rat model and MMQ cells to explore whether TBMA and hordenine could alter DRD2 or its methylation status.

## Materials and Methods

### Human Subjects

This study was comprised of 46 schizophrenic patients taking Ris for a long time. The study has received ethical approval from Pharmacy Department of Wuhan University Tongren Hospital (The Third Hospital of Wuhan). The patients were divided into high PRL level (HPRL) and normal PRL level (NPRL) groups. Normal PRL levels were 2.64–13.13 ng/ml in males and 3.34–26.72 ng/ml in premenopausal females (<50 years). The demographic data of the subjects are provided in Table 1. The mean age of the participants is 29.24 ± 2.57. Venous blood samples (200 μl) were collected from the subjects, and genomic DNA was extracted. The DNA solutions were kept at −80°C prior to analysis.

**TABLE 1:** Demographic and clinical characteristics of the two groups.

	HPRL (*n* = 24)	NPRL (*n* = 22)	*p*
Females (n/%)	17 (70.8)	13 (59%)	0.465
Age (years)	31.3 ± 1.26	28.11 ± 3.76	0.154
BMI (kg·m^−2^)	20.45 ± 2.16	21.35 ± 5.1	0.732
PRL level before Ris (ng/mL^−)^	16.32 ± 4.39	19.44 ± 5.16	0.231
PRL level after Ris (ng·mL^−1^)	83.21 ± 11.19	21.56 ± 1.77	0.00018***
Ris dose (mg/day)	3.53 ± 0.97	2.16 ± 1.01	0.79
Smoking (years/%)	2 (8)	5 (22.7)	0.452
Drinking (years/%)	1 (4)	2 (9)	0.112
Psychiatric history (years/%)	16 (66.7)	13 (59)	0.543

Note: Values denote the mean ± standard deviation.

### Chemicals and Reagents

The following chemicals and reagents were used in this study: raw maiya (batch number: 20200501, Hubei Pingpong Hongkang Traditional Chinese Medicine Decoction Pieces Co., Ltd., China); TBMA [prepared using a previously established TBMA extraction and purification method (21)], purity: 65.7%; hordenine [C_10_H_15_NO, MW:165.24, Aladdin Reagent (Shanghai) Co., Ltd., China], purity: ≥99%; Bro (C_33_H_44_BrN_5_O_8_S, MW: 750.7, MedChemExpress, United States), purity: >99.98%; Ris (batch number: KFB6W00A, Xian Janssen Pharmaceutical Co., Ltd., China.); 5-Aza-2′deoxycytidine (C_8_H_12_N_4_O_4_, MW:228.21, MedChemExpress, United States), purity: 99.93%.

### Animal Model

Specific-pathogen-free (SPF) female Sprague Dawley (SD) rats (200 ± 20 g) were purchased from the Experimental Animal Center of Three Gorges University [Hubei, China; Grant No. SYXK(E)2020–0080]. The experimental protocol was approved by the Ethics Committee of The Third Hospital of Wuhan. All animal experiments complied with the Animal Research: Reporting of *In Vivo* Experiments guidelines and were conducted according to the National Institutes of Health Guide for the Care and Use of Laboratory Animals (NIH Publications No. 8023, revised 1978).

### Animal Groups and Drug Administration

Chronic treatment with Ris (0.1 mg/kg/day) causes increased PRL levels in Sprague-Dawley rats (Wang et al., 2018). Bro is a well-known dopamine agonist that suppresses pituitary PRL release in MMQ cells and improves antipsychotic-induced PRL levels in rats (Miranda and Jones, 2007; Bernard et al., 2015; Wei et al., 2017; Zhou et al., 2018; Bernard et al., 2019). It served as a positive control for this study. Seventy rats were randomly assigned into seven treatment groups as follows: 1) blank control, 2) Ris, 3) Ris + Bro (5 mg/kg), Ris + Hor (5 mg/kg), Ris + N-Methyltyramine (N-Methy) (5 mg/kg), Ris + TBMA (3.6 mg/kg), Ris + TBMA (7.2 mg/kg) (*n* = 10). Ris (0.1 mg/kg) was administered orally at 9:00 a.m. The control group was given a 5% gum arabic solution. Bro, Hor, N- Methy, and TBMA were given orally to their corresponding groups 2 h after Ris administration. Treatment with Ris and the other agents was performed once daily for 8 weeks. At week 7, fasting blood was collected. At week 8, all animals were fasted and then euthanized by cervical dislocation under anesthesia. The pituitary glands were collected, flash-frozen in liquid nitrogen, and stored at −80°C.

### Cell Culture and Treatment Groups

MMQ rat pituitary tumor cells were purchased from Beijing Beina Chuanglian Biotechnology Institute. These cells secrete prolactin and express dopamine receptors and are, thus, an ideal *in vitro* cell model system for our study. The cells (1 × 10^5^) were seeded in 6-well plates. After 24 h, the cells were the treated with Bro, Hor, or TBMA for 72 h. The DNMT inhibitor 5′-AZA was also used to establish the DNMT low-expression cell model.

### Enzyme-Linked Immunosorbent Assay

Blood samples were collected retro-orbitally from the rats. The PRL ELISA kit was purchased from LunChangShuo Biotech (Cat. # SU-B30285, Xiamen, China).

### Real-Time Fluorescence Quantitative PCR

Total RNA was extracted from the frozen pituitary samples using the Total RNA Kit (batch number: R6934010000C17U031, Omega). cDNA was synthesized using the SweScript RT I First Strand cDNA Synthesis Kit with gDNA Remove). mRNA expression was measured using the Stratagene Mx 3000P Real-Time PCR system (Agilent Technologies, CA, United States). Relative expression was calculated using the 2^−ΔΔCt^ method. All values were normalized to the expression of the housekeeping gene GAPDH. The primer sequences are listed in Table 2.

**TABLE 2:** The sequences of the real-time PCR primers.

Gene	Sequence
GAPDH	Forward primer: 5′- GAC​ATG​CCG​CCT​GGA​GAA​AC-3′
	Reverse primer: 5′- AGC​CCA​GGA​TGC​CCT​TTA​GT-3′
PRL	Forward primer: 5′- GGT​TTG​GTC​ACA​ACT​CCC​ATC​CC -3′
	Reverse primer: 5′- TGG​ACA​ATT​TGG​CAC​CTC​AGG​AAC -3′
DRD2	Forward primer: 5′- AAG​ACG​ATG​AGC​CGC​AGA​AAG​C -3′
	Reverse primer: 5′- AGC​AGA​TGA​TGA​ACA​CAC​CGA​GAA​C -3′
DNMT1	Forward primer: 5′- TGT​TCC​TCC​TTC​TGC​CAT​CAA​TGT​G -3′
	Reverse primer: 5′- CAT​CGT​CCT​TAG​CGT​CGT​CGT​AAC -3′
DNMT3α	Forward primer: 5′- CGT​CAC​ACA​GAA​GCA​TAT​CCA​GGA​G -3′
	Reverse primer: 5′- CAG​GAG​GCG​GTA​GAA​CTC​AAA​GAA​G -3′
DNMT3β	Forward primer: 5′- GAT​GGA​GAT​GGT​GAA​GCG​GAT​GAT​G -3′
	Reverse primer: 5′- AGG​CTG​GAG​ATA​CTG​TTG​CTG​TTT​C -3′

### Western Blot Analysis

PRL, DRD2, and DNMTs protein levels in the pituitary samples were determined by western (WB) blot analysis, as previously described (Gong et al., 2021). The antibodies used in this experiment were anti-PRL (1:6000, Affinity, United States), anti-DRD2 (1:1000, Wuhan Proteintech Co., Ltd., China), anti-DNMT1 (1:1000, Wuhan Proteintech Co., Ltd., China), anti-DNMT3α (1:1000, Affinity, United States), and anti-DNMT3β (1:1000, ABclonal, China).

### MassARRAY Sequencing

MassARRAY sequencing was used to detect the methylation rate of DRD2. The CpG island of the DRD2 promoter region was sequenced by Huada Gene Technology Co., Ltd. (Beijing, China).

### Statistical Analysis

Data are presented as the mean ± standard deviation (SD; normal distribution). Differences between groups were analyzed with the one-way analysis of variance (ANOVA) comparison test using SPSS 22.0 software (SPSS, Chicago, IL, United States). A *p*-value < 0.05 was considered statistically significant.

## Results

### Comparison of DRD2 Methylation Rates Between HPRL and NPRL Subjects

**FIGURE 1:**
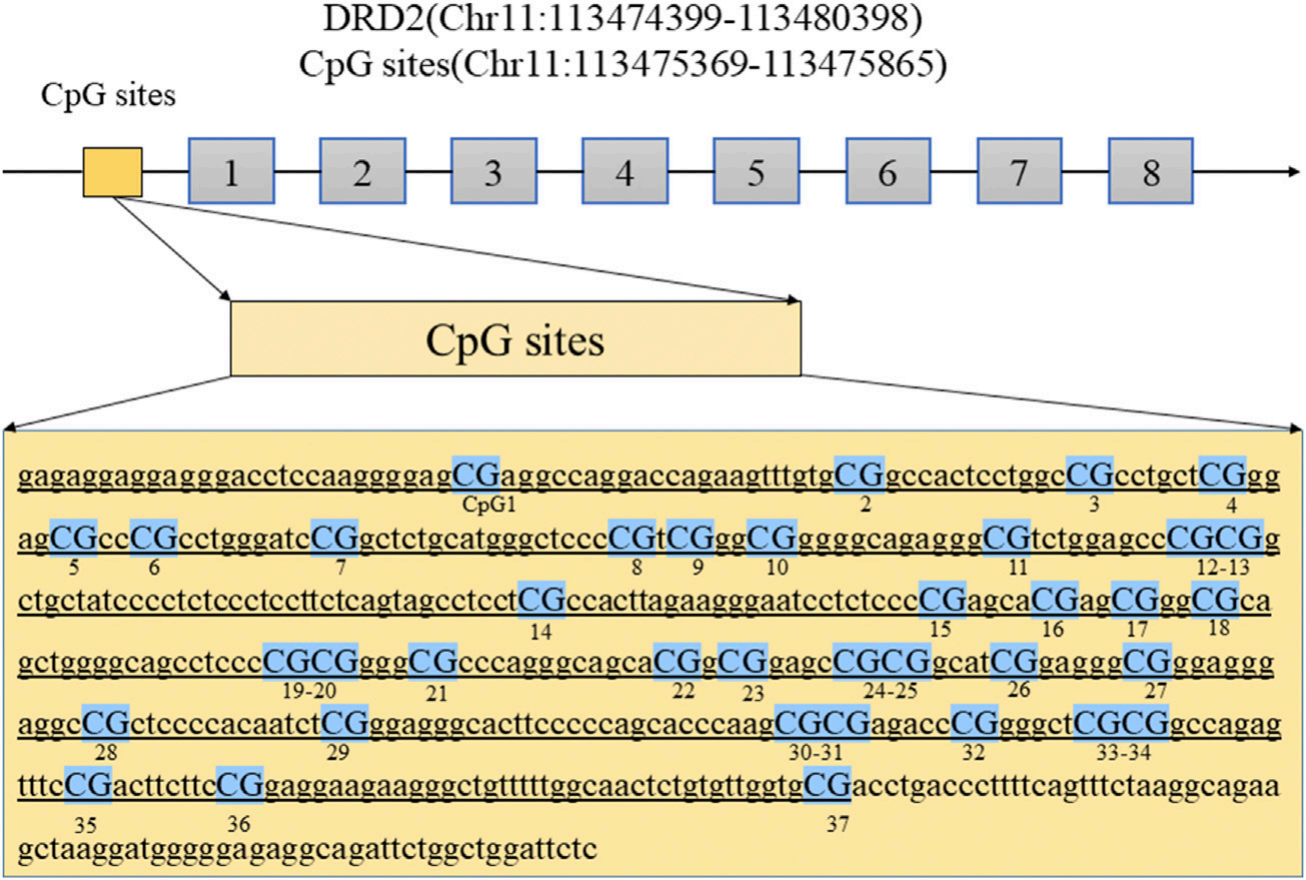
The diagram shows the location of the DRD2 promoter regions analyzed in this study. Abbreviations: DRD2, dopamine receptor D2 gene.

**FIGURE 2:**
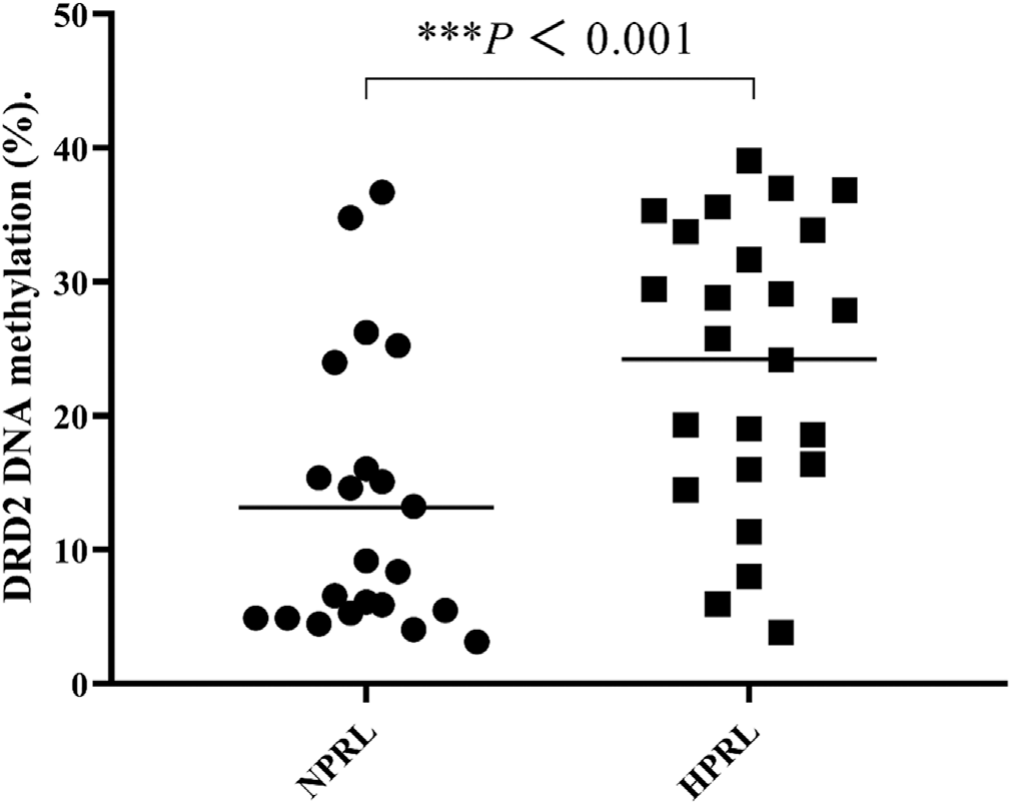
Comparisons of DRD2 DNA methylation (%) between HPRL and NPRL subjects. Each bar represents the mean ± standard error (^***^ *p* < 0.001 versus NPRL).

Figure 1 illustrates the structure of the DRD2 promoter region with the CpG sites for methylation that may play a central role in gene silencing (Figure 1). The demographic data for each group of subjects are shown in Table 1. HPRL and NPRL subjects did not differ in sex, age, or BMI (*p* > 0.05). The results of the DRD2 methylation analysis following long-term risperidone monotherapy are shown in Figure 2. The positive rate of DRD2 methylation in the HPRL group was significantly higher than in the NPRL group (*p* < 0.001).

### Effects of TBMA and Hordenine on PRL in Ris Induced the PRL Level Increased Rats

**FIGURE 3:**
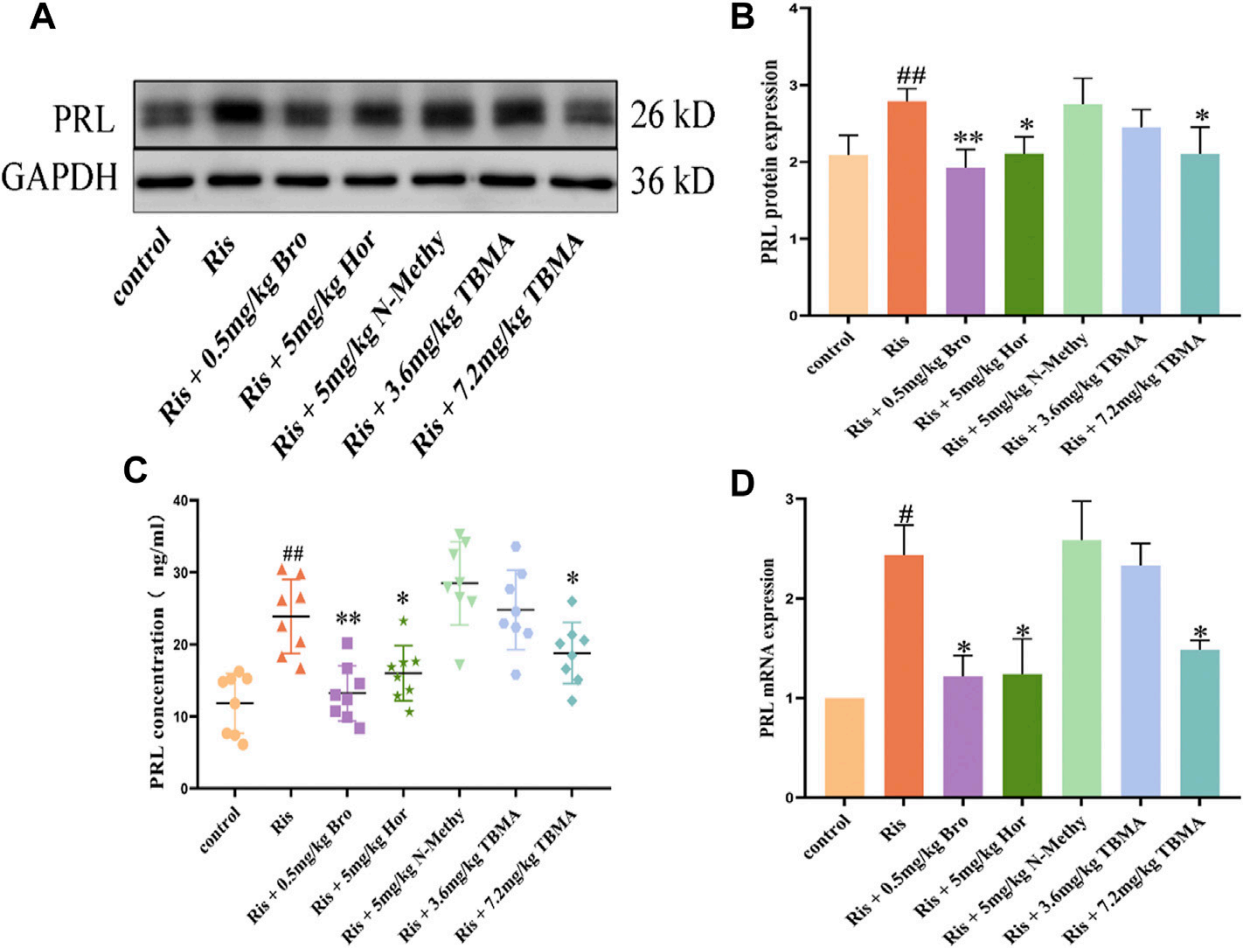
Effects of TBMA, hordenine, and N-methyltyramine on PRL expression. **(A,B)** PRL protein expression determined by western blotting using GAPDH as a loading control. Data are presented as the mean ± SD (*n* = 3 rats per group). **(C)** PRL concentrations detected by ELIA (*n* = 8). **(D)** PRL mRNA expression. mRNA expression was determined using RT-PCR (*n* = 3 rats per group; ^*#*^ *p* < 0.05 and ^*##*^ *p* < 0.01 versus the control group; ^*^ *p* < 0.05 and ^**^ *p* < 0.01 versus the Ris group. Bro = bromocriptine; Hor = hordenine; N-Methy = N-methyltyramine; Ris = risperidone; TBMA = total barley maiya alkaloids.

Based on the clinical results, we established a rat model of elevated PRL levels by long-term administration of Ris to female rats. These rats were then treated with vehicle, Bro, Hor, N-Methy, or 3.6 mg/kg or 7.2 mg/kg TBMA. The ELISA results showed that Ris increased the fasting serum PRL concentration, and Hor and 7.2 mg/kg TBMA decreased the Ris-induced PRL concentrations (Figure 3C). After treatment with Bro, hordenine, or 7.2 mg/kg TBMA, PRL protein (Figures 3A,B) and mRNA (Figure 3D) expression levels were significantly decreased in the pituitary. In contrast, N-methyltyramine and 3.6 mg/kg TBMA did not affect PRL expression.

### TBMA and Hordenine Reversed the Changes in PRL Levels Induced by Ris Concomitant With Decreased DRD2 Expression and Increased DNMTs Expression in the Pituitary

**FIGURE 4:**
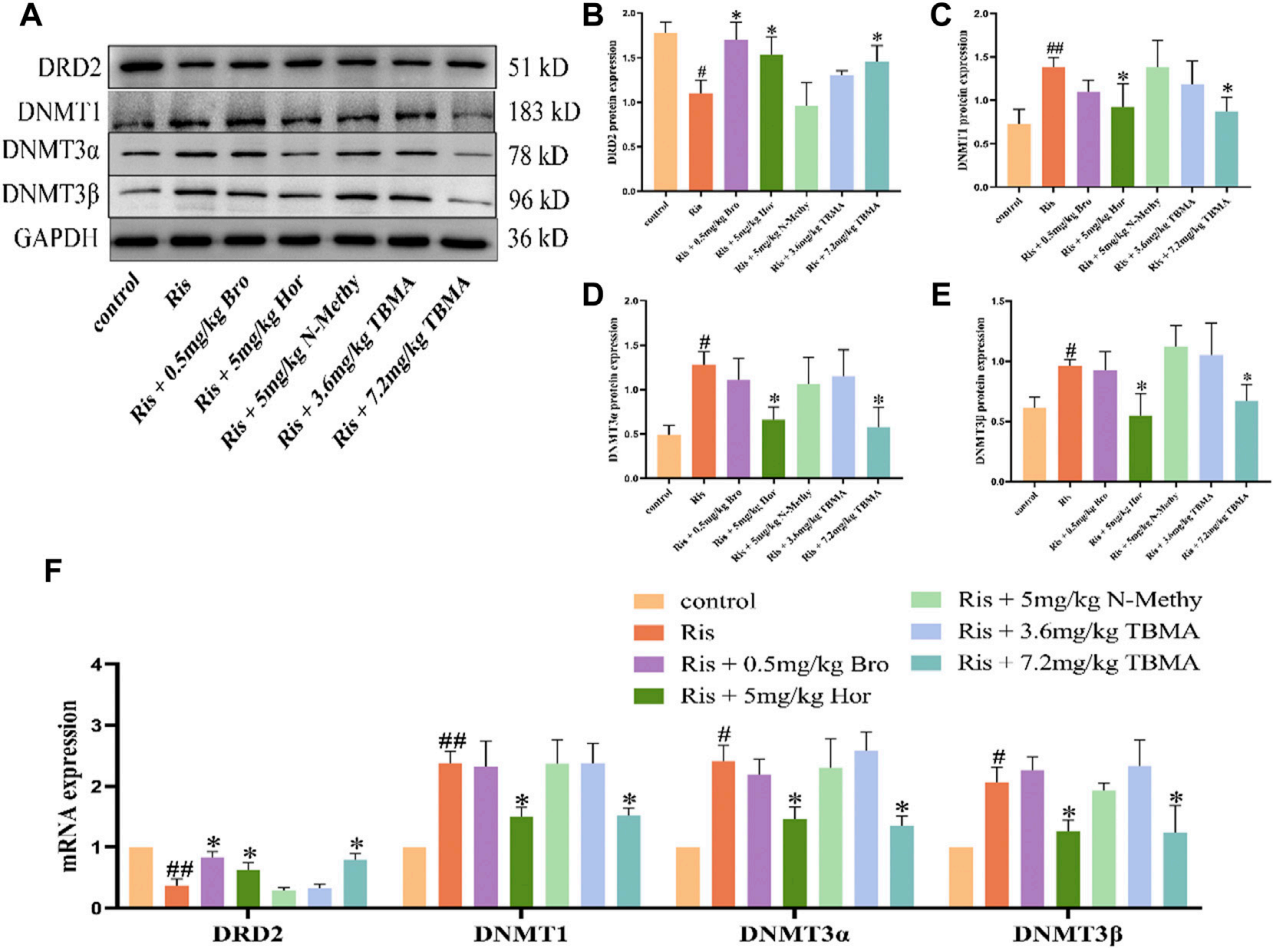
Effects of TBMA, hordenine, and N-methyltyramine on DRD2 and DNMT expression. **(A–E)** DRD2, DNMT1, DNMT3α, and DNMT3β protein expression. GAPDH was used as a loading control. **(F)** DRD2, DNMT1, DNMT3α, and DNMT3β mRNA expression. Data are presented as the mean ± SD. Protein expression in each group was determined using western blotting. mRNA expression in each group was determined using RT-PCR (*n* = 3 rats per group; ^*#*^ *p* < 0.05 and ^*##*^ *p* < 0.01 versus the control group; ^*^ *p* < 0.05 versus the Model group. Bro = bromocriptine; Hor = hordenine; N-Methy = N-methyltyramine; Ris = risperidone; TBMA = total barley maiya alkaloids.

We examined the changes in DRD2 and DNMTs expression in each rat group. DRD2 mRNA expression was downregulated in the Ris group, whereas DNMT1, DNMT3α, and DNMT3β mRNA expression levels were upregulated. Compared to the Ris group, the DRD2 mRNA expression levels in the Ris + Hor (5 mg/kg) group and Ris + TBMA (7.2 mg/kg) group were significantly upregulated, while the mRNA expression levels of the DNMTs were significantly downregulated. Compared with the Ris group, the DRD2 mRNA expression was also significantly upregulated in the Ris + Bro (0.5 mg/kg) group; however, DNMT mRNA expression did not change. Moreover, DRD2 and DNMT mRNA expression did not change in the Ris +5 mg/kg N-methyl and Ris + TBMA (3.6 mg/kg) group (Figure 4F). The WB analysis results were consistent with the mRNA results (Figures 4A–E). Ris-induced PRL levels could reduce DRD2 levels and increase DNMT mRNA and protein levels in the rat pituitary. Furthermore, hordenine and 7.2 mg/kg TBMA could restore DRD2 levels and reduce DNMT expression. Bro could also increase DRD2 levels. N-methyl and 3.6 mg/kg TBMA had no significant effects on DRD2 or the DNMTs.

### DRD2 Methylation Was Significantly Upregulated by Ris and Downregulated by TBMA and Hordenine

**FIGURE 5:**
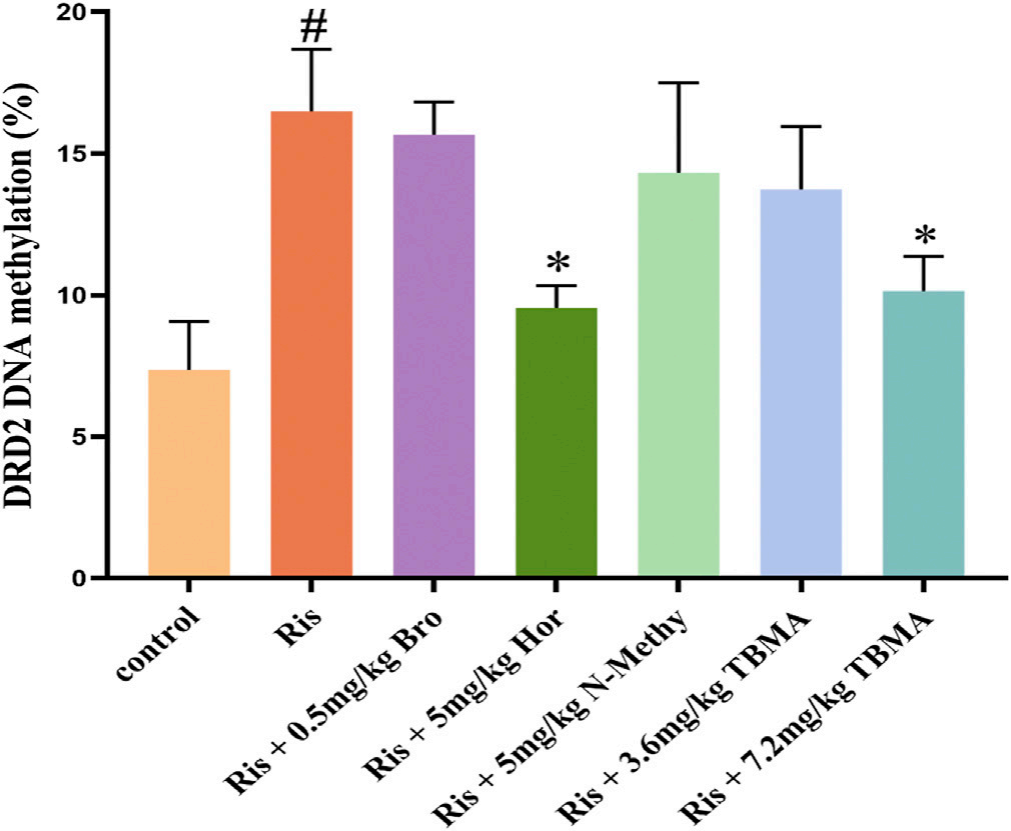
DRD2 methylation status. Data are presented as the mean ± SD (*n* = 3 per group). (^*#*^ *p* < 0.05 versus the control group; ^*^ *p* < 0.05 versus the Ris group). Bro = bromocriptine; Hor = hordenine; N-Methy = N-methyltyramine; Ris = risperidone; TBMA = total barley maiya alkaloids.

To clarify the mechanism underlying decreased DRD2 expression, we investigated whether DRD2 promoter hypermethylation was responsible for this downregulation using MassARRAY sequencing. Ris treatment enhanced DRD2 promoter methylation while significantly decreasing DRD2 expression. In contrast, 7.2 mg/kg TBMA and hordenine upregulated DRD2 expression levels by causing DRD2 promoter demethylation. However, Bro, N-methyltyramine, and 3.6 mg/kg TBMA did not have this effect. Together, these results suggest that the decreased DRD2 expression observed with Ris-induced increased PRL levels resulted from reversible methylation of the DRD2 promoter region (Figure 5).

### TBMA and Hordenine Protected MMQ Cells From Ris-Induced PRL Increased Levels and DRD2 Methylation

**FIGURE 6:**
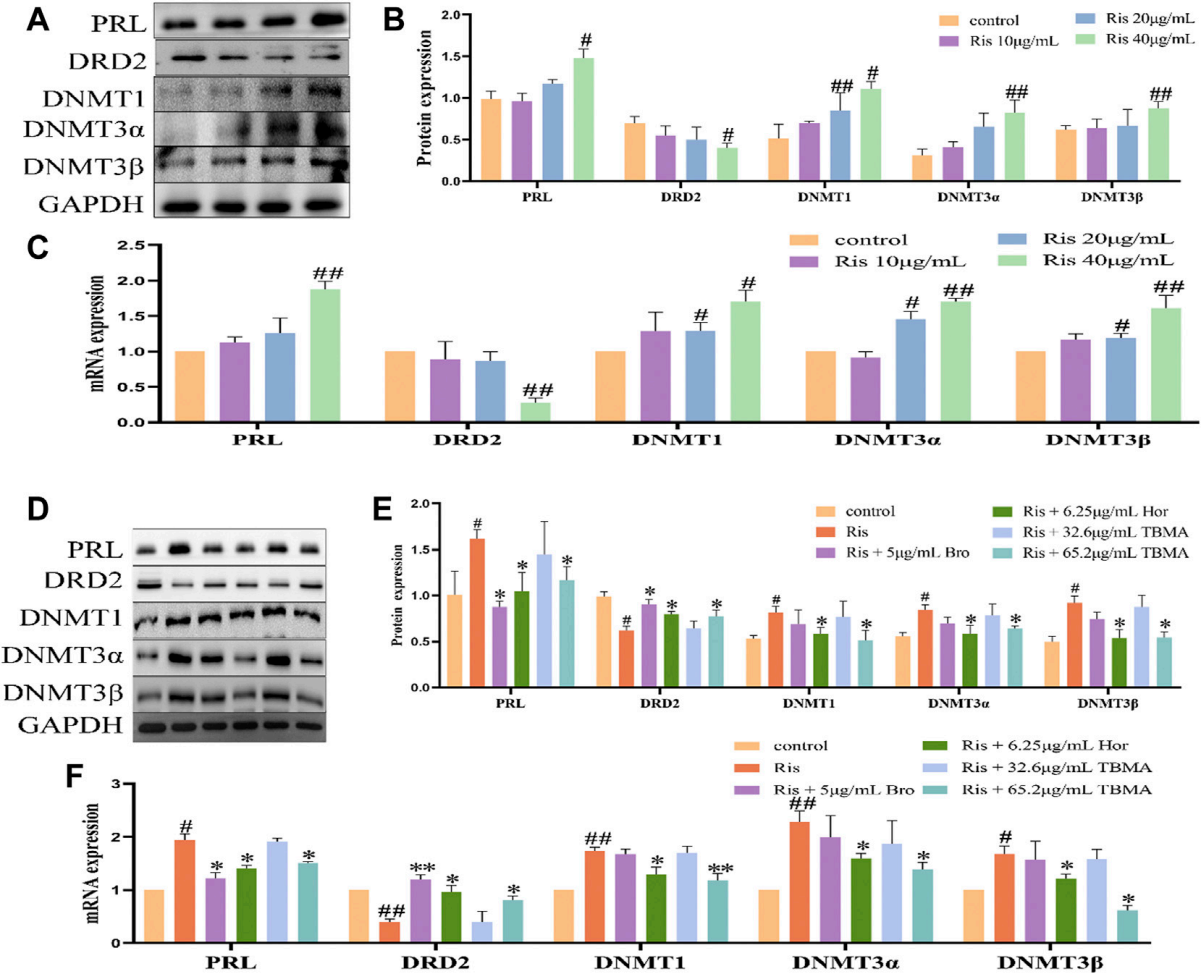
TBMA and hordenine regulated DRD2 DNA methylation. **(A,B)** PRL, DRD2, and DNMT protein expression by western blotting following treatment with different risperidone concentrations (*n* = 3). **(C)** PRL, DRD2, and DNMT mRNA expression levels by RT-PCR following different risperidone concentrations (*n* = 3). **(D,E)** WB was used to detect PRL, DRD2, and DNMT protein expression by western blotting following TBMA or hordenine treatment of MMQ cells (*n* = 3). **(F)** was used to detect the mRNA levels of PRL, DRD2, and DNMT mRNA expression by RT-PCR following TBMA or hordenine treatment of MMQ cells (*n* = 3). Data are presented as the mean ± SD (^*#*^ *p* < 0.05 and ^*##*^ *p* < 0.01 versus the control group; *p < 0.05 and **p < 0.01 versus the Ris group). Hor = hordenine; Ris = risperidone; TBMA = total barley maiya alkaloids.

To verify the results obtained with the clinical samples and in rats, we further evaluated the effects of Ris, TBMA, and Hor *in vitro* using MMQ cells. Because N-methyltyramine had no effect *in vivo*, only TBMA and hordenine were studied *in vitro*. In this experiment, Ris (10, 20, and 40 μg/ml) was used to induce PRL levels in the MMQ cells. We found that 40 μg/ml Ris increased PRL expression, decreased DRD2 expression, and upregulated the expression levels of DNMT1, DNMT3α, and DNMT3β (Figures 6A–C). Treatment with 65.2 μg/ml TBMA or 6.5 μg/ml hordenine abrogated the effects of Ris on PRL, DRD2, and the DNMTs. The lower TBMA concentration had no effect (Figures 6D–F). These data were consistent with the rat data demonstrating that TBMA and hordenine could inhibit PRL expression by reversing DRD2 methylation induced by Ris.

### The Effects of TBMA and Hordenine on DRD2 Methylation and PRL Expression Was Enhanced by Modulating DNMT Expression

**FIGURE 7:**
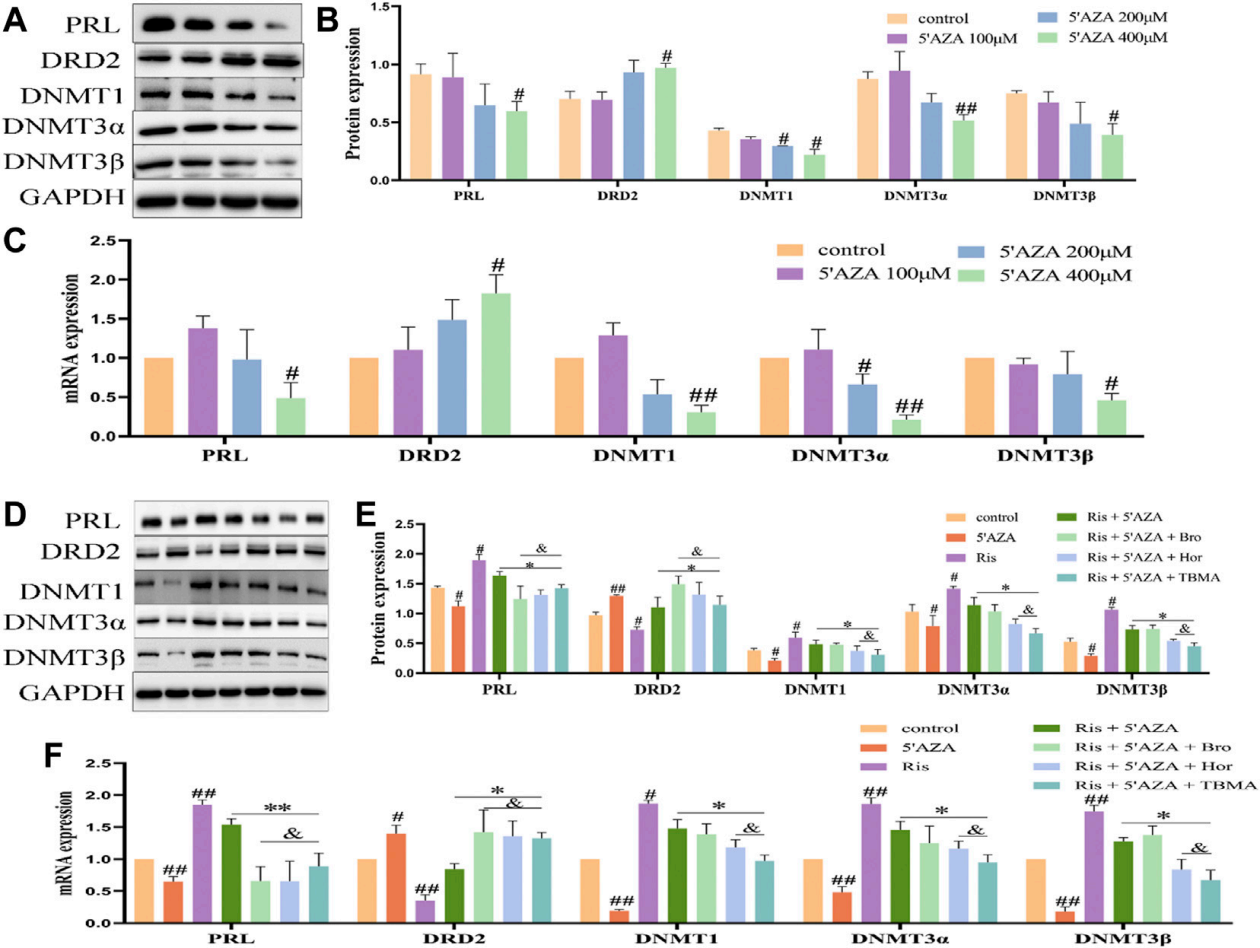
The effects of TBMA and hordenine on DRD2 DNA methylation were enhanced by low DNMT expression levels. **(A,B)** PRL, DRD2, and DNMT protein expression by western blotting following treatment with different 5′AZA concentrations (*n* = 3). **(C)** PRL, DRD2, and DNMT mRNA expression by RT-PCR following treatment with different 5′AZA concentrations (*n* = 3; *p < 0.05 and **p < 0.01 versus the control group. **(D,E)** PRL, DRD2, and DNMT protein expression by western blotting following TBMA or hordenine treatment of MMQ cells (*n* = 3). **(F)** PRL, DRD2 and DNMT mRNA expression by RT-PCR following TBMA or hordenine treatment of MMQ cells (*n* = 3). Data are presented as the mean ± SD (^*#*^ *p* < 0.05 and ^*##*^ *p* < 0.01 versus the control group; *p < 0.05 and **p < 0.01 versus the Ris group; ^*&*^ *p* < 0.05 versus the Ris +5′AZA group. 5′AZA = 5-Aza-2′-deoxycytidine; Hor = hordenine; Ris = risperidone; TBMA = total barley maiya alkaloids.

5′AZA is an inhibitor of DNA methyltransferase. Treatment of MMQ cells with different concentrations of 5′ AZA (100 μM, 200 μM, and 400 μM) revealed that 400 μM 5′AZA significantly inhibited the expression of DNMT1, DNMT3α, and DNMT3β while increasing DRD2 levels and inhibiting PRL expression (Figures 7A–C). Therefore, 400 μM 5′AZA was selected for the subsequent experiment. Compared to the Ris +5′AZA group, the mRNA and protein expression levels of PRL and the DNMTs were decreased in the Ris +5′AZA +65.2 μg/ml TBMA and Ris +5′AZA +6.5 μg/ml Hor groups and DRD2 expression levels were increased (Figures 7D–F), further demonstrating that TBMA and hordenine could enhance DRD2 expression and decrease PRL expression through demethylation.

## Discussion

This study investigated the correlation between DRD2 methylation and the ability of TBMA and hordenine to modulate PRL levels increased by ADs. We found that the positive rate of DRD2 methylation in HPRL patients was significantly higher than in NPRL patients after long-term administration of Ris. Ris is a DRD2 antagonist that could reduce DRD2 expression in our studies, indicating that DNA methylation could increase PRL expression by reducing DRD2 expression. Previous studies demonstrated that TBMA and hordenine could inhibit PRL expression by restoring the expression of DRD2 (Gong et al., 2021). Our current study further revealed that TBMA and hordenine could increase DRD2 expression and reduce PRL levels through DRD2 promoter demethylation *in vivo* and *in vitro*. The methylation of specific genes has been associated with transcriptional inactivation (Suzuki and Bird, 2008). Therefore, DRD2 hypermethylation is consistent with the observed decrease in DRD2 expression.

ADs cause abnormal increases in PRL levels (Grigg et al., 2017; Molitch, 2020), which seriously affect the quality of life of patients. To overcome these increased PRL levels, patients are administered bromocriptine or have their dose of ADs reduced and combined with aripiprazole (Rusgis et al., 2021). However, bromocriptine has many adverse side effects, including neurological and digestive system disorders (Rusgis et al., 2021). Reducing the dose of ADs can lead to the recurrence of a patient’s condition and affect treatment efficacy. When combined with aripiprazole, it will increase the burden of cognitive function in patients (Jiang et al., 2021). Therefore, the development of new drugs that reduce the increased PRL levels caused by ADs is needed to improve the quality of life of schizophrenic patients.

Epigenetic modifications are most commonly regulated by direct methylation of DNA or post-translational modifications of histones, both of which can promote or repress gene transcription (Asa and Ezzat, 1998; Yoshino et al., 2007). Epigenetic dysregulation, promoter methylation, and silencing of tumor suppressor genes are implicated in pituitary neoplasia (Asa and Ezzat, 1998). Pituitary tumorigenesis often involves genetic mutations in classical oncogenes or tumor suppressor genes (Asa and Ezzat, 2002; Thumfart et al., 2021). DRD2 methylation is closely related to children’s psychological trauma (Cespedes et al., 2021) and alcohol dependence (Shirvani-Farsani et al., 2021) and is a marker of schizophrenia (Gangisetty et al., 2015; Molitch, 2020; Lisoway et al., 2021) showed that increased DRD2 promoter methylation is correlated with decreased DRD2 mRNA levels and increased PRL mRNA levels in the pituitary. Many studies have reported that the loss of DRD2 expression accelerates the increase in PRL levels caused by ADs (Gao et al., 2021). As an important active component of TBMA, hordenine has demonstrated agonistic effects on DRD2 improving DRD2 expression levels may be a significant approach to preventing AD-induced increases in PRL levels (Gong et al., 2021).

DNA methylation provides a new therapeutic approach for targeted inhibition of tumor factors and a possible way to overcome cancer drug resistance (Robertson, 2001). Current knowledge regarding the genetic and epigenetic regulation of DRD2 is mainly restricted to the oncology field (Tan et al., 2021). As such, this study is the first to investigate whether DRD2 methylation is involved in the underlying mechanisms of AD-induced increases in PRL levels. We detected the methylation rate of DRD2 in the pituitary of rats with increased PRL levels following treatment with the AD Ris. We found that the methylation rate in the CpG island of the DRD2 promoter region was increased by Ris but could be reversed by TBMA or hordenine treatment. We also found that the expression levels of DNMT1, DNMT3α, and DNMT1β were increased in rats with elevated PRL levels and Ris-treated MMQ cells. The DNMTs are responsible for transferring methyl groups from s-adenosine methionine to the 5′-position of cytosine residues in DNA (Stresemann et al., 2006). In particular, DNMT1 acts as a maintenance methyltransferase, whereas DNMT3α and DNMT3β are *de novo* methyltransferases (Edwards et al., 2017). Thus, our study revealed that increased DNMT expression might lead to CpG methylation in the DRD2 promoter.

5′-AZA is a nucleoside-based DNMT inhibitor that induces demethylation and gene reactivation. It is a cytosine analog, which is metabolically activated *in vivo* and readily incorporated into DNA during replication (Christman, 2002). We used 5′-AZA to verify the role of DNMT-mediated methylation in regulating DRD2 expression. We found that 5′AZA caused demethylation of the DRD2 promoter and a significant increase in DRD2 expression, consistent with our hypothesis. By decreasing the expression of the DNMTs with 5′AZA, the protective effects of TBMA and hordenine were attenuated. Together, these results confirmed that TBMA and hordenine could improve DRD2 expression and reduce the secretion of PRL by inhibiting its expression mediated by the DNMTs, which is consistent with another study demonstrating that increased DRD2 promoter methylation in the rat pituitary is closely related to decreased DRD2 mRNA levels and increased PRL mRNA levels.

In this study, we found that the positive rate of DRD2 methylation in HPRL patients was significantly higher than in NPRL patients after long-term administration of Ris at the clinical level. Our current study revealed that TBMA and hordenine could increase DRD2 expression and reduce PRL levels through DRD2 promoter demethylation *in vivo* and *in vitro*. However, only 46 patients with schizophrenia were included in the clinical study, which is a small number of cases, so the sample size should be expanded for further study. which will be the focus of our future research.

We also observed the interesting phenomenon that TBMA had a greater effect on DNMT levels than hordenine, suggesting that other unknown components in TBMA can decrease the expression of the DNMTs. In future TBMA studies, we hope to identify these other components and further characterize the specific components regulating DRD2 methylation.

## Conclusion

Our study clearly indicates that TBMA could reduce the incidence of AD-induced PRL level increases through DRD2 methylation, providing new insights into the epigenetic mechanism of the increased PRL levels caused by chronic AD administration and potential new treatment strategies based on epigenetic regulation. These findings could lay a foundation for future research and clinical intervention. Indeed, clarifying the biological mechanism mediating the relationship between DRD2 methylation and increased PRL levels can bring new and personalized prevention and treatment possibilities, reduce pain, and improve the quality of life.

## Data Availability

The raw data supporting the conclusion of this article will be made available by the authors, without undue reservation.
